# Quality control and safety assessment of BCG vaccines in the post-genomic era

**DOI:** 10.1080/13102818.2014.927200

**Published:** 2014-07-10

**Authors:** Tzvetelina Stefanova

**Affiliations:** ^a^ Bul Bio – National Center of Infectious and Parasitic Diseases (BB-NCIPD) Ltd., Sofia, Bulgaria

**Keywords:** BCG vaccine, quality control methods, genomic characterization

## Abstract

A hundred and five years ago, Albert Calmette and Camille Guérin began a daunting task, which is unmatched even today, that led to the most widely used vaccine in human history. Despite a century of scientific advances, BCG (an acronym for Bacillus Calmette–Guérin) remains the only vaccine for prevention of tuberculosis. Due to the fact that the use of BCG vaccines will continue, either as a stand-alone or as a prime vaccine in prime-boost immunization strategies, the World Health Organization (WHO) has underlined the necessity for further work toward better characterization, evaluation and quality control of the BCG vaccine, taking into account recent advances in genetics and molecular biology. The potential benefit of such improved characterization could be addressed to better and easier differentiation between sub-strains used by different manufacturers. It may help to ensure consistency of production in terms of genetic stability and it may also help the clinical evaluation of new antituberculosis vaccines. Last but not least, the state-of-the-art technologies could facilitate the quality control performed by the manufacturers and by National Control Authorities as well.

## Introduction

The BCG (Bacillus Calmette–Guérin) live, attenuated vaccine is the only vaccine used for immunoprophylaxis of tuberculosis since 1921 till now. BCG is the most widely administered vaccine in the world, with 91.7% global coverage in 2012.[1] Till now more than 3 billion people have been immunized all over the world. Despite few discrepant data about its protective efficacy, there is no reliable alternative to the BCG vaccine and this vaccine will continue to be used as a golden standard during the process of new antituberculosis vaccine development.

A number of unresolved issues in the quality assessment of BCG vaccine have been identified [2–4] and still exist. These include immunological mechanisms of efficacy and protection induced by different BCG vaccines; genetic differences of sub-strains and their implications to genotype characteristics; immunogenicity and efficacy in animal models and in humans and also, the impact of vaccine characteristics on the safety profile of different products. In terms of quality control a key issue is lack of correlates of protection and therefore the absence of a tool to distinguish protective from non-protective vaccines.[4] Furthermore, the current quality control test methods for BCG vaccine have limited potentialities as they were developed many years ago.

Due to the fact that the use of BCG vaccines will continue, either as a stand-alone or as a prime vaccine in prime-boost immunization strategies, the necessity for further work in better characterization of the BCG vaccines and of the production strains has been underlined.[4] The importance of product characterization needs to be strongly emphasized as well-defined vaccines offer the greatest chance of success.

The World Health Organization (WHO) has recognized the need to improve both characterization of this vaccine and the assays used for its control, taking into consideration recent advances in genetic and molecular biology. The potential benefit of such improved characterization could be addressed to better and easier differentiation between sub-strains used by different manufacturers; it may help to ensure consistency of production in terms of genetic stability and also this may help the clinical evaluation of BCG vaccines in the future.[2–4]

A better understanding of vaccine characteristics relevant to production and control as well as to the safety and efficacy in humans should lead to improved control tests. For this purpose relevant and effective vaccine evaluation using state-of-the-art technologies is of fundamental importance for testing BCG immunogenicity, BCG safety, BCG identity and potency and last but not least – in searching of immune correlates in clinical trials (for new vaccines).

The following quality control tests could be a subject to improvement and could be potentially replaced by new and more perfect models in agreement with current scientific achievements.

## Potency testing of BCG vaccine by modified ATP assay for viable count

The BCG vaccine is based on a live attenuated strain of *Mycobacterium bovis*. The viability of organisms is essential for the stimulation of a protective immune response and monitoring viable counts is an integral part of quality control. Although measurement of viable cells is not in itself an assay of potency, it has been used as a surrogate of BCG immunogenicity and efficacy.[4–8]

However, the cultural viable count assay, often known as colony-forming unit (CFU) test, is problematic and very time consuming, as mycobacteria are very slow growing microorganisms.[4,9] Due to the slow growth of the organisms, a 4–5 weeks’ incubation period is needed to complete the assay. Additionally, the results from the CFU test can be variable and often not reproducible because of inherent difficulties associated with mycobacterial cultivation, including tendency of these microorganisms to clump and their requirement for complex growth media. The difficulties associated with this time- and labour-consuming and variable assay impact the BCG manufacturing processes such as formulation of final product from the bulk substance and assessments of stability. The slowness, poor reproducibility and high variability of test results are the main driving forces for manufacturers and control laboratories to look for a rapid, more reproducible alternative procedure for quantifying BCG culturable particles.[9,10] This issue has been addressed in recent WHO consultation meetings on characterization and improvement of the quality control of BCG vaccines.

Since adenosine triphosphate (ATP) is a major metabolite of living cells which is rapidly lost in dead cells, measuring ATP content can provide reliable estimates of the number of living cells. The determination of ATP based on bioluminescence is one of the alternative methods for rapid detection of the viability of the BCG vaccine.[11,12] Bioluminescence is the production and emission of visible light as a result of biochemical reactions where chemical energy is converted into light. Most bioluminescent reactions are caused by oxidation or oxygenation.

A modified ATP bioluminescence assay has been developed by Statens Serum Institute (SSI) as an alternative rapid assay for viable count of BCG vaccine.[10] This ATP assay is based on the reaction of firefly luciferase with ATP which results in a bioluminescent product. The reaction is accompanied by light emission.[13,14] The intensity of light emission measured is directly proportional to ATP content in the sample which can be estimated by using the ATP standards for calibration. High correlation has been observed between intracellular ATP concentration and the number of viable BCG bacilli in different vaccine preparations. Furthermore, the results from an international collaborative study initiated by WHO indicated that the ATP bioluminescence assay is an easy to perform, robust and reproducible method that could be routinely used as a quality control procedure in the manufacture of BCG vaccine.[9] Another main advantage of this method is that it enables evaluation of extremely low metabolic concentrations.

The modified ATP assay is simple and easy to perform. The test is rapid and time saving; the time to obtain results is reduced from five weeks to three days. The method is by far more reproducible than the cultural method. The sensitivity of the bioluminescent method is much higher than the one of traditional methods such as spectrophotometric and fluorometric assays. There is a high range of linearity between the values of the registered signal and the concentration of the evaluated substance. Other advantages of this method are that small amounts of the examined sample are used, and also simple and not expensive equipment.

However, future studies should be carried out on the modified ATP assay in order to establish the reproducibility and suitability of this method. These investigations should be advanced by further examinations of the correlation between the cultural viable counts and ATP content of different BCG vaccines produced. It is important for each individual laboratory to establish this correlation by experimental tests for each BCG product.

## Improvement of the identity test: identification of BCG vaccine by multiplex PCR

The number of sub-strains used for BCG vaccine production has effectively been reduced to five: Russian BCG-I, Tokyo 172-1, Danish 1331, Moreau RDJ and Pasteur 1173-P2. These five sub-strains account for more than 90% of the BCG production worldwide. Since the current identity test for BCG vaccine using acid-fast staining and colony morphology lacks specificity, it is essential to develop a robust, routine assay for manufacturers and regulatory laboratories to identify different sub-strains of BCG. This will also ensure genetic consistency in production, from master seed lot through working seed lot and to final product. Different approaches such us DNA microarray to look for single nucleotide polymorphisms (SNPs), deletions and duplications can provide a better insight into the molecular characterization of different BCG sub-strains. Although these techniques are not intended for routine use during the production, they can identify regions of interest and importance for further studies. For the purpose of the last revision of WHO recommendations for BCG production and control,[15] the improvement of the identity test was further considered with respect to the methodology to be used as well as to factors that may influence variability of sub-strains during the production of vaccines.

The current required identity test for BCG vaccine is acid-fast staining together with a characteristic appearance of colonies grown on solid medium. These microbiological techniques for testing BCG identity cannot distinguish *M. bovis* BCG from other bacteria in the *Mycobacterium tuberculosis* complex, most of which are virulent. This main disadvantage calls for searching for more reliable methods which can differentiate BCG sub-strains themselves as well as distinguish BCG sub-strains from pathogenic mycobacteria. Now, the monograph for BCG in European Pharmacopoeia has stated that molecular techniques may be used as alternatives for identification.

One of the alternatives for BCG identity testing is the multiplex PCR (mPCR) assay, which was developed in the National Institute for Biological Standards and Control (NIBSC), UK.[16] As a result of the sequencing of the mycobacterial genome,[17] it has now been possible to perform comparative genomics of BCG vaccine by whole-genome DNA microarray. Using this method, 16 regions (RD1–RD16) were found to have been deleted from BCG strains in relation to the virulent strain of *M. tuberculosis* H37Rv. Some deletions vary between BCG sub-strains.[18] As summarized in [19], RD1 was lacking from all BCG vaccines and is presumed to have been lost during the initial attenuation between 1908 and 1921. The deletion of RD2 is thought to have occurred at the Institut Pasteur between 1927 and 1931. Another deletion, RD14, which is specific to BCG Pasteur, occurred between 1938 and 1961. Further deletions that seem to have occurred away from the Institut Pasteur are the loss of RD8 and RD16. These five regions have been successfully exploited to produce a fingerprint that differentiates between sub-strains. In addition to those five targets, the SenX3-RegX3 mycobacterial two-component system (responsible for the virulence of *M. tuberculosis*, and for phosphate-dependent gene expression) has also been identified as a successful target site for use in identifying BCG sub-strains.[19] The mPCR method has been evaluated in an international collaborative study to assess its accuracy, robustness and reproducibility for use as an identity test for BCG vaccine.[16] It has been demonstrated that the mPCR assay is highly specific and able to identify and differentiate among BCG sub-strains and to distinguish between both these strains and *M. tuberculosis*.[20] The assay has proved to be robust with consistent and reproducible results across a number of laboratories.

The method is suitable not only for comparing and identification of BCG sub-strains in manufacture and control (especially in changing Seed lot), but also for specific identification of BCG isolates from a variety of clinical situations including both immunosuppressed children and adults undergoing therapy for bladder cancer.

Once standardized within a control laboratory, the novel mPCR could be very effective for determining the identity of BCG vaccines.[19]

## *In vitro* approaches in testing absence of virulent mycobacteria

In the 2004 WHO consultation report, guinea pigs are reported to be currently used for routine monitoring of the presence of virulent mycobacteria in BCG vaccine.[2] This assay is very time consuming as the animals are under observation over six weeks after injection of BCG vaccine. As an alternative, a PCR-based *in vitro* assay has been developed [21] which can detect DNA specific or not to virulent mycobacteria. It is well known that all BCG vaccines have deletions in the RD1 region which affect both the *esat-6* and the *cfp-10* gene. PCR targeting these two genes allowed discrimination between BCG and pathogenic mycobacteria and thus – detection of contamination. The sensitivity of this assay ranged from 1 genome (equivalent to 1 fg DNA) for *cfp-10* primers and 1000 genomes for *esat-6* primers when using purified DNA preparations.

Further work is required, as pointed out in the 2004 WHO report,[2] to apply this PCR assay for assuring freedom from *M. tuberculosis* contamination in BCG vaccines. Applying this new method will not only reduce the time required for testing absence of virulent mycobacteria in BCG products, but also significantly reduce the use of animals and therefore, a lot of ethical considerations can be solved.

## Genetic characterization of BCG vaccines

A recent advance that permits a more rigorous analysis of BCG strains has been the sequencing of the entire genome of *M. tuberculosis.*[17] The assembling of whole genome DNA microarray representing 99.4% of predicted genes [18] demonstrates not only differences between BCG and *M. tuberculosis* but also differences among BCG vaccines. This fact reveals ongoing evolution of BCG strains after 1921. Each sub-strain has its own signature molecular profile and differs from other sub-strains. These differences can be used to characterize any sample of BCG vaccine.[3]

Current BCG sub-strains are genomically different from one another. Apart from deletion of genomic regions (RDs), tandem duplications (DU1, DU2) and SNPs have also been observed in different sub-strains. In addition, variability in gene expression profiles and at transcriptomic level among sub-trains is also under investigation.[2] All these specific characteristics could be applied for further BCG evaluation in a general way.

The most appropriate schedule for genetic typing of BCG suggested in the 2005 WHO report is based on variable number tandem repeat (VNTR) number at MIRU 4, RD pattern and number and location of tandem duplications.[4] This scheme was successfully used to characterize BCG strains recovered from patients with complications of vaccinations. It could be also used for monitoring the consistency of BCG production.

In parallel, it is important to know how genetic differences are expressed in the final product. Identification of differences in protein expression offer a way towards this, as there is accumulating knowledge about proteins relevant to the protective response to tuberculosis. Using the proteomic technique, it is possible to identify the different protein profiles of different vaccine strains. This is particular important to identify any changes in protein profiles from working seed, different passage levels to final lot preparation of the product.

Currently, as a part of the BCG vaccine characterization programme, extensive molecular genetic characterization studies have been undertaken. As a result, the data about whole sequencing and deciphering the genome of *M. bovis* BCG were published.[22]

The results about comparative genome and transcriptome analysis revealed extensive variations in gene expression between early BCG strains (Japan, Birkhaug and Russia) and late BCG strains (Pasteur, Danish and Glaxo). The variations result from increased gene dosage or from altered activity of pleiotropic regulators leading to over- or under-production of certain proteins, including virulence factors and enzymes.[22] Brosch et al. [22] have also reported that there are extensive differences in the level of expression of known surface proteins and immunodominant protein antigens between early and later BCG sub-strains that may induce protective responses. The same team established an evolutionary scheme for BCG vaccine by analysing different genetic makers and uncovered that the variability affects gene expression levels, immunogenicity and possibly, protection against tuberculosis. Furthermore, the combined findings suggest that better protection against tuberculosis may be conferred by early BCG vaccines,[22] which could be considered to include BCG Sofia (descended from BCG Russia).

Molecular typing and genome sequencing of *M. bovis* BCG, sub-strain Sofia SL222, gave an insight to the specific genetic properties of the Bulgarian BCG vaccine.[23] The genetic profile from master seed lot, working seed to final lot was investigated. *M. bovis* BCG, sub-strain Sofia, has two copies of IS 6110. The second copy is inserted into the promotor region of the gene *phoP* which increase the gene expression and induce the higher level of virulence. One of the main characteristics of the strain is the presence of RD2 region, which is typical only for the ‘early’ BCG sub-strains (Moreau, Tokyo, Russia). RD2 encompasses the region Rv1979c-Rv1989c (11 genes; 8.9 kb); including gene *mpt64* coding the high-immunogenicity protein МРТ64. Another specificity of the strain is a rearrangement in the genome – a DU2 type I duplication in the region Rv3299-Rv3316 encoding enzymes of the citric acid cycle. This duplication generates genome plasticity, which indicates that the genome is still dynamic. A novel 1.6 kb deletion was identified that affects the genes Rv3697c and Rv3698, related to membrane proteins in the cell wall structure. This region is also deleted in the genome of BCG Russia but not in any other strain. This deletion therefore must have occurred prior to the *in vitro* cultivation of BCG Sofia.

The genome of BCG Sofia SL222 was compared to those of sub-strains Tokyo 172, Pasteur 1173P2, *M. bovis* AF 2122/97 and *M. tuberculosis* H37Rv. The genome of BCG Sofia SL222 was shown to be closer to that of Tokyo 172 (99.68% similarity), rather than to Pasteur 1173-P2. The size of the genome of *M. bovis* BCG Sofia SL222 is 4 369 629 bp and contains 4317 genes functionally distributed into 309 sub-systems.

As a result of the genetic characterization of *M. bovis* BCG Sofia it is now clear that the strain harbours genetic properties which pertain only to the strains closest to the original one of Calmette and Guérin. The genome of BCG Sofia (BCG Russia, respectively) appears to be most conservative among the genomes of other ‘early’ BCG sub-strains, which contributes to its genetic stability. Thus, one of the most powerful enabling technologies of the post-genomic era proved the genetic identity, provenance and stability of the Bulgarian BCG vaccine for a period longer than 30 years.

## Conclusions

The genotyping of BCG vaccines has an important value in standardization and differentiation of sub-strains used in vaccine manufacture. It can prove the consistency of production regarding its genetic stability. In addition, last but not least, the molecular characterization of BCG sub-strains unfolds an opportunity for finding reliable correlates in clinical assessment of protective efficacy and safety of the BCG vaccine in humans, which is particularly important in the development of new antituberculosis vaccines or immunization strategies. 
